# Responses in species diversity in the Hulunbuir grassland to phosphorus addition under nitrogen-limiting and non-limiting conditions

**DOI:** 10.3389/fpls.2024.1393471

**Published:** 2024-07-17

**Authors:** Zhihui Wang, Li Chen, Yuzhen Pan, Dan Zhao, Yunrui Yang, Xinyu Li, Hongyi Wang

**Affiliations:** ^1^ College of Horticulture and Landscape Architecture, Heilongjiang Bayi Agricultural University, Daqin, China; ^2^ Key Laboratory of Low-carbon Green Agriculture in Northeastern China, Ministry of Agriculture and Rural Affairs, Daqing, China; ^3^ Agricultural Products and Processed Products Supervision and Testing Center, Ministry of Agriculture, Daqing, China

**Keywords:** nitrogen deposition, phosphorus addition, nitrogen limitation, species diversity, functional group

## Abstract

The phenomenon of nitrogen deposition resulting in species loss in terrestrial ecosystems has been demonstrated in several experiments. Nitrogen (N) and phosphorus (P), as major nutrients required for plant growth, exhibit ecological stoichiometric coupling in many ecosystems. The increased availability of nitrogen can exacerbate the ecological effects of phosphorus. To reveal the ecological effects of phosphorus under nitrogen-limiting and non-limiting conditions, we conducted a controlled N–P interaction experiment over 5 years in the Hulunbuir meadow steppe, where two nitrogen addition levels were implemented: 0 g N·m^-2^·a^-1^ (nitrogen-limiting condition) and 10 g N·m^-2^·a^-1^ (nitrogen-non-limiting condition), together with six levels of phosphorus addition (0, 2, 4, 6, 8, and 10 g P·m^-2^·a^-1^). The results showed that nitrogen addition (under nitrogen-non-limiting conditions) significantly decreased species diversity in the steppe community, which was exacerbated under phosphorus addition. Under nitrogen-limiting conditions, phosphorus addition had no marked impact on species diversity compared to the control; however, there were substantial differences between different levels of phosphorus addition, exhibiting a unimodal change. Under both experimental nitrogen conditions, the addition of 6 g P·m^-2^·a^-1^ was the threshold for affecting the community species diversity. Nitrogen addition reduced the relative biomass of legumes, bunch grasses, and forbs, but substantially increased the relative biomass of rhizomatous grasses. In contrast, phosphorus addition only markedly affected the relative biomass of forbs and rhizomatous grasses, with the former showing a unimodal pattern of first increasing and then decreasing with increasing phosphorus addition level, and the latter exhibiting the opposite pattern. The different responses of rhizomatous grasses and other functional groups to nitrogen and phosphorus addition were observed to have a regulatory effect on the changes in grassland community structure. Phosphorus addition may increase the risk of nitrogen deposition-induced species loss. Both nitrogen and phosphorus addition lead to soil acidification and an increase in the dominance of the already-dominant species, and the consequent species loss in the forb functional group represents the main mechanism for the reduction in community species diversity.

## 1 Introduction

Since the Industrial Revolution, anthropogenic nitrogen deposition in terrestrial ecosystems has rapidly increased, becoming a critical environmental issue of the 21st century (Galloway et al., 2008). Grasslands, an essential component of terrestrial ecosystems, play a crucial role in maintaining food security and biodiversity (Fang et al., 2018). The increase in available nitrogen has impacted the structure and function of grassland ecosystems, including the mitigation of nitrogen limitation, enhancement of primary productivity, and increase in carbon sequestration in grasslands, while also leading to the loss of species diversity and reducing the stability of such ecosystems (Seabloom et al., 2021; Band et al., 2022). Excessive nitrogen inputs alter the proportion of nutrients available to plants, shifting plants originally limited by nitrogen to being limited by phosphorus or co-limited by nitrogen and phosphorus (Peñuelas et al., 2013). As one of the essential elements for plant growth, phosphorus plays a vital role in energy transfer and photosynthesis (Jiang et al., 2019). Therefore, studying the ecological effects of phosphorus in the context of nitrogen deposition can elucidate the impacts of nitrogen deposition on the structure and function of grassland ecosystems.

Species diversity is a critical basis for ecosystem services and constitutes an important indicator for grassland restoration and ecosystem management (Tilman, 1993). Competition for resources resulting from environmental changes (e.g., nutrient enrichment) often leads to alterations in grassland species diversity, i.e., increases or decreases in community diversity (Bai et al., 2010; Zhang et al., 2022). The niche dimensionality hypothesis posits that the increase in limiting resources reduces the dimensionality of niches, thereby intensifying interspecific competition and leading to a decrease in species diversity (Harpole et al., 2016). In contrast, the theory of functional trait diversity suggests that species-specific asymmetric competition can increase community species diversity (Niu et al., 2014). The impact of exogenous nitrogen input on grassland species diversity is currently a topic of great interest in the field of global change (Yang et al., 2022a, 2022b). Early nitrogen deposition surveys in British grasslands showed that an annual input of 2.5 kg·hm^-2^ within a 2 × 2 m plot could cause species loss (Stevens et al., 2004). Multi-level nitrogen addition experiments conducted on the Qinghai–Tibet Plateau and the Inner Mongolia grassland in China also confirmed that increased availability of N leads to a reduction in species diversity (Zhang et al., 2014; Ma et al., 2021). The main mechanism was soil acidification, light competition, ammonium toxicity and litter accumulation caused by nitrogen addition, all contributing to a decrease in community species diversity (Nihlgård, 1985; Hautier et al., 2009; Stevens et al., 2010). According to the theory of ecological stoichiometric homeostasis, nitrogen and phosphorus in plants often exhibit a coupling relationship (Yu et al., 2010). The increase in nitrogen deposition also exacerbates the ecological effects of phosphorus. However, current studies have not reached a consensus on the impact of phosphorus on grassland species diversity (Jiang et al., 2019).

Grassland plant communities are composed of multiple species that play different ecological roles in maintaining community stability (Wang et al., 2020a). In grassland ecology research, communities are often classified into functional groups based on ecological traits, such as categorizing grassland communities into rhizomatous grasses, leguminous plants, and forbs (Bai et al., 2010). Species within the same functional group exhibit similar responses to environmental disturbances. In nutrient addition experiments, Tian et al. (2022) found that nitrogen addition had no marked effect on Gramineae and Cyperaceae, but substantially reduced the functional group diversity in forbs; long-term low-level nitrogen addition experiments at Cedar Creek also revealed a loss of rare species (Clark and Tilman, 2008). Phosphorus benefited the functional groups in leguminous plants and forbs but had no substantial impact on the diversity of Gramineae species (Wang et al., 2020b). The mechanisms of the impact of nitrogen on different functional groups in grasslands are relatively clear; in contrast, the effects of phosphorus on different functional groups in grasslands require further extensive research.

The Hulunbuir Grassland is experiencing rapid increases in nitrogen deposition (Yang et al., 2012), which necessitates accurate prediction of ecological effects to enable sustainable grassland utilization. Our study, based at the Erguna Forest–Steppe Ecotone Research Station of the Chinese Academy of Sciences, investigates the effects of phosphorus on grassland plant communities and functional group species diversity under nitrogen-limiting and non-limiting conditions. Our research aims to explore the related mechanisms and their impacts on species diversity and to provide scientific technical support and well-founded suggestions for grassland ecological management.

## 2 Materials and methods

### 2.1 Overview of the experimental area

This study was conducted in Hulunbuir City, Inner Mongolia Autonomous Region, at the Erguna Forest–Steppe Ecotone Ecosystem Research Station of the Chinese Academy of Sciences (50°10′46′′ N, 119°22′56′′ E). The region is characterized by a cold, temperate, continental, monsoon climate, with an average annual rainfall of 360 mm and an average temperature of -2.5°C. The soil type is dark calcareous soil, and the vegetation is primarily composed of Gramineae, Asteraceae, Rosaceae, and Leguminosae. The community is dominated by *Leymus chinensis*, *Stipa baicalensis*, *Thermopsis lanceolata*, and *Cleistogenes squarrosa*, accompanied by *Cymbaria dahurica*, *Potentilla tanacetifolia*, *Heteropappus altaicus*, and *Carex duriuscula*. Since its enclosure in 2013, the area has experienced minimal human disturbance, except for experimental treatments and sampling.

### 2.2 Experimental design

The experiment was conducted at the Nitrogen and Phosphorus Addition Control Experimental Platform of the station. The platform was enclosed in 2013 (soil baseline data: organic carbon 20.53 g·kg^-1^; total nitrogen content 1.81 g·kg^-1^; total phosphorus content 0.47 g·kg^-1^) and treatments began in 2014. Fertilization was performed annually in May before plant growth resumed, using a dry application method for all fertilizers. Nitrogen and phosphorus fertilizers were applied as potassium dihydrogen phosphate (KH_2_PO_4_) and ammonium nitrate (NH_4_NO_3_), respectively. Two nitrogen addition levels were set: 0 and 10 N·m^-2^·a^-1^ (designated as N0 and N10, respectively), with N0 assumed to represent a nitrogen-limiting condition and N10 a nitrogen-non-limiting condition. Six levels of phosphorus addition were implemented: 0, 2, 4, 6, 8, and 10 g P·m^-2^·a^-1^. This resulted in a total of 12 treatments with five replications, yielding 60 plots in total. Each plot measured 8 × 8 m, arranged in a completely randomized block design. Based on habitus and the nitrogen-fixing characteristics of leguminous plants, the grassland plant community was divided into four functional groups (Bai et al., 2010): leguminous plants (LE), perennial rhizome grasses (PR), perennial bunch grasses (PB), and perennial forbs (PF).

### 2.3 Sampling and index calculation

In mid-August 2018, during the period of maximum plant biomass, a field quadrat survey was conducted, with each quadrat measuring 1 × 1 m. Aboveground plants were clipped and counted by species and the samples were conveyed to the laboratory. They were dried at 65°C for 48 h, after which the biomass was weighed and calculated for each species. Soil sampling was conducted concurrently with the quadrat survey, consisting of the collection of 0–10 cm of topsoil to test for inorganic nitrogen, available phosphorus, pH value, and other parameters.

Species diversity indices were calculated according to Sun et al. (1993). The calculation formulas were:

(1) Species richness (SR)


SR=S

(2) Shannon-Wiener diversity index (H’)


$$H^{\prime }=-\sum_{i=1}^{S}P_{i}ln\;P_{i}$$

(3) Simpson’s dominance index (D)


$$D=1-\sum P_{i}^{2}$$

where S represents the number of species in a 1 × 1 m quadrat; *i* represents the *i*-th species in the community; and *P_i_* is the relative biomass of the *i*-th species.

### 2.4 Data analysis

Data pre-processing was conducted using MS Office (2021) and statistical analysis was performed using SPSS 21.0. We used one-way analysis of variance (ANOVA) to evaluate the differences between treatments (significance level, *P<* 0.05; Duncan’s test), and two-way ANOVA to determine the effects of nitrogen, phosphorus, and their interaction on soil pH, available nutrients, community biomass, community species diversity, different functional group relative biomass, and species richness. Pearson and Mantel tests were used for correlation analysis to ascertain the relationships between various indices. Graphs were produced using Graphpad Prism 7 software.

## 3 Results

### 3.1 Soil pH and available nutrients

**Figure 1:**
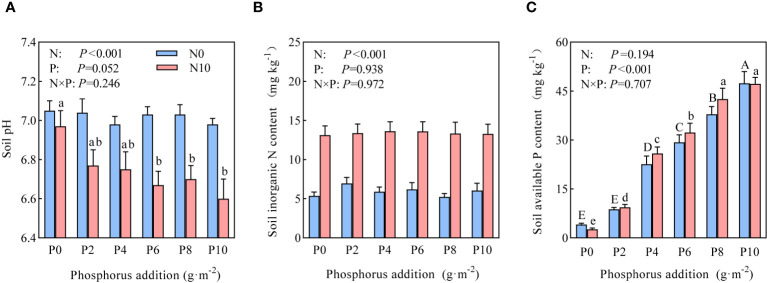
Effects of nitrogen and phosphorus addition on **(A)** soil pH, **(B)** inorganic nitrogen, and **(C)** available phosphorus. Different uppercase and lowercase letters represent significant differences at *P<*0.05.

Two-way ANOVA revealed that nitrogen substantially affected soil pH and inorganic nitrogen content, whereas phosphorus had a substantial impact on available phosphorus content (**Figure 1**). Under the N0 treatment (nitrogen-limiting), phosphorus addition had no marked effect on soil pH, whereas it substantially reduced soil pH under the N10 treatment (nitrogen-non-limiting) in parallel with increasing addition amounts (**Figure 1A**). The inorganic nitrogen content in the soil under the N10 treatment substantially increased to nearly 50% of that of the N0 treatment (**Figure 1B**), but there was no statistical differences between the phosphorus addition treatments. Progressive phosphorus addition substantially increased the content of soil available phosphorus (**Figure 1C**).

### 3.2 Impact of nitrogen and phosphorus addition on community biomass and species diversity

**Figure 2:**
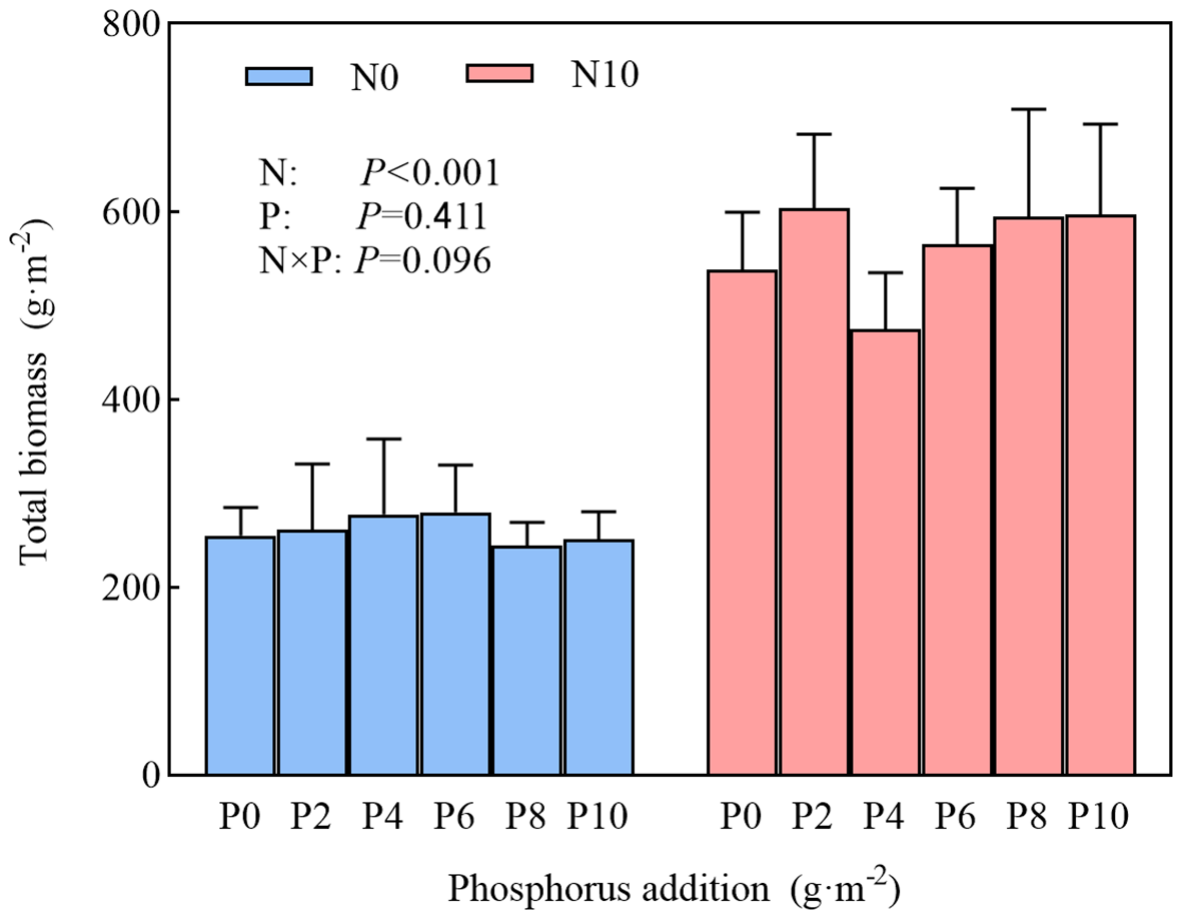
Effects of nitrogen and phosphorus addition on grassland plants community biomass.

**Figure 3:**
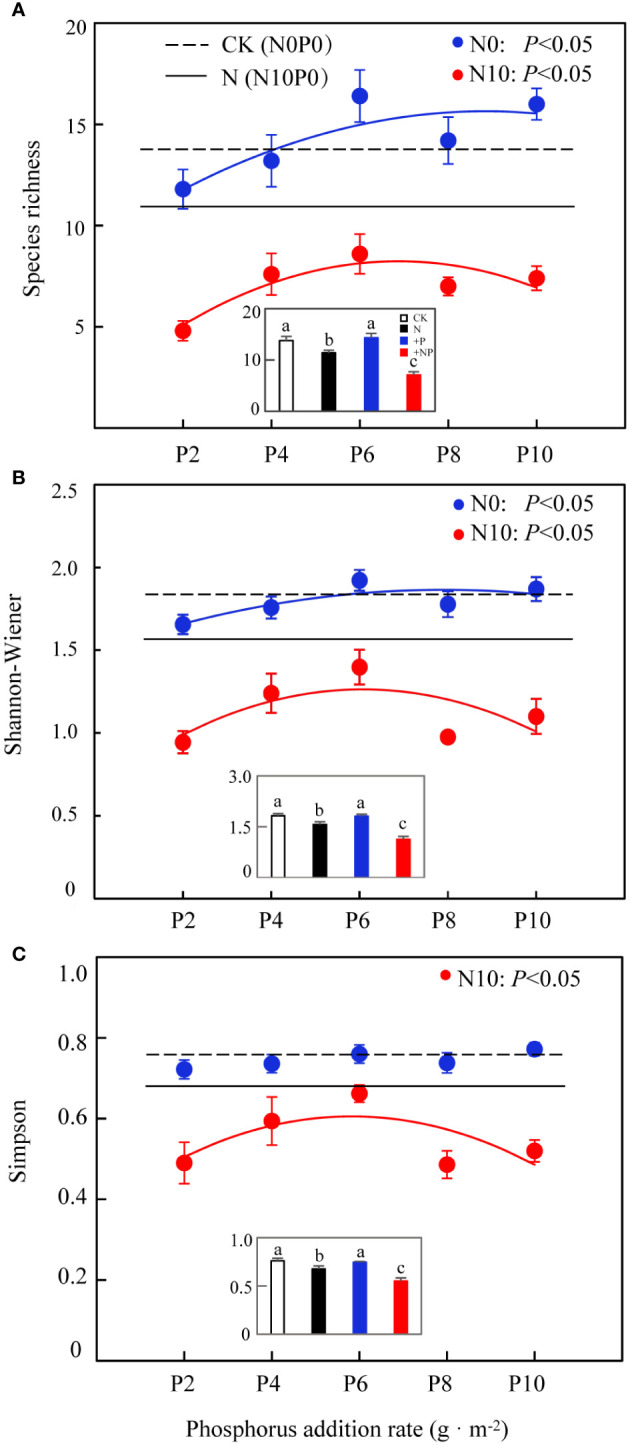
Effects of nitrogen and phosphorus addition on grassland plants community species diversity. **(A)** Species richness, **(B)** Shannon–Wiener index, **(C)** Simpson index. CK (inset): N0P0; N (inset): N10P0; +P (inset): average value of phosphorus-addition-only treatment; + NP (inset): average value of combined nitrogen and phosphorus addition treatment. Different lowercase letters indicate significant differences at *P<* 0.05.

A higher nitrogen content substantially increased total community biomass, whereas phosphorus and the interaction of nitrogen and phosphorus had no marked effect on this metric, and there were no substantial differences among different levels of phosphorus addition (**Figure 2**). In N10, community species diversity was substantially reduced (**Figure 3**; **Table 1**), and this reduction was more pronounced with the simultaneous addition of nitrogen and phosphorus (**Figures 3A–C** insets). In N0, phosphorus addition had no marked impact on community species diversity (**Figures 3A–C** insets), but there were marked differences among different levels of phosphorus addition (except for the Simpson’s index). Nitrogen addition substantially reduced community species richness, as indicated by the average species richness under nitrogen addition and under combined nitrogen and phosphorus addition being significantly lower than CK (**Figure 3A** insets). In both N0 and N10 treatments, community species richness showed a unimodal curve across different levels of phosphorus addition, increasing initially and then decreasing after a threshold at 6 g·m^-2^. The Shannon–Wiener diversity index of the community exhibited a similar pattern, changing unimodally with phosphorus addition (**Figure 3B**). Nitrogen addition substantially reduced the Simpson’s index, which also showed a unimodal curve under the combined addition of nitrogen and phosphorus, with the average Simpson’s index being substantially lower than CK. (**Figure 3C**, insets).

**Table 1:** Effects of nitrogen and phosphorus addition on grassland plants community species diversity (two-way ANOVA).

Treatment	Simpson		Shannon-Wiener		Community richness	
	F	P	F	P	F	P
N	64.53	<0.001	78.504	<0.001	82.22	<0.001
P	3.144	<0.05	3.378	<0.05	3.478	<0.01
N×P	2.095	0.082	1.717	0.149	1.740	0.144

Tabulated F and P values for comparison between groups.

### 3.3 Impact of nitrogen and phosphorus addition on the relative biomass and richness of different functional group species

**Figure 4:**
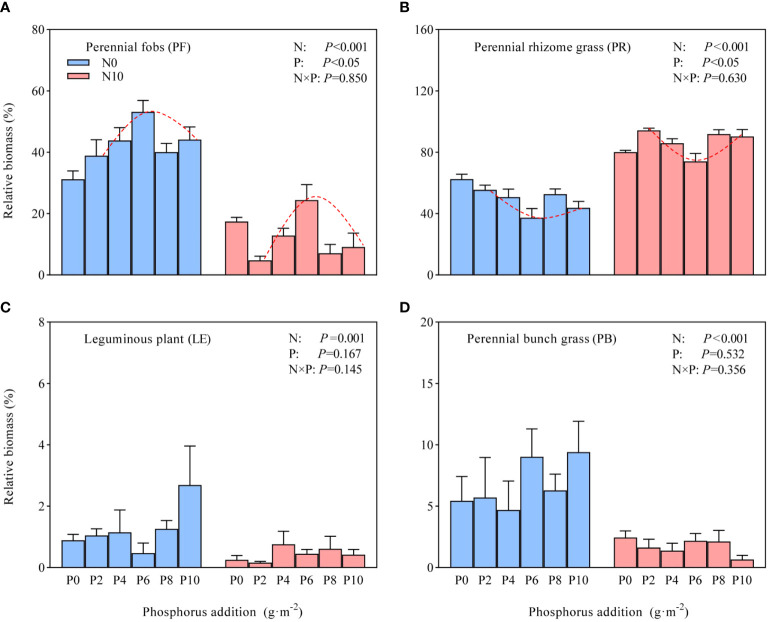
Effects of nitrogen and phosphorus addition on different functional group relative biomass (mean ± standard error, SE). **(A)** perennial forbs, **(B)** perennial rhizome grasses, **(C)** leguminous plants, **(D)** perennial bunch grasses. N, nitrogen addition; P, phosphorus addition.

Nitrogen addition substantially affected the relative biomass of forbs, rhizomatous grasses, leguminous plants, and bunch grasses (**Figure 4**). In contrast, different levels of phosphorus addition only substantially affected the relative biomass of forbs and rhizomatous grasses, with no statistical interaction between nitrogen and phosphorus. Due to interspecies competition for resources and the different nutrient preferences of species in various functional groups, there were considerable changes in the relative biomass of each functional group across treatments. In N10, all treatments reduced the relative biomass of legumes, bunch grasses, and forbs, with average reductions of 64.80%, 74.41%, and 69.90%, respectively, while increasing the relative biomass of rhizomatous grasses by an average of 70.58%. Thus the increase in relative biomass of rhizomatous grasses dominated the changes in community biomass under nitrogen-non-limiting conditions. The biomass of forbs exhibited a unimodal curve, initially increasing and then decreasing with increasing phosphorus addition (except for N10P0), whereas rhizomatous grasses showed the opposite pattern, decreasing initially and then increasing (again except for N10P0) (**Figures 4A, B**). The changing trends in the relative biomass of these two functional groups indicate that at the approximate addition of 6 g P·m^-2^, the biomass of forbs reached a turning point in its increase in both N0 and N10, with excessive addition leading to a decline in their importance in the community, whereas rhizomatous grasses occupied the growth space left by forbs.

**Figure 5:**
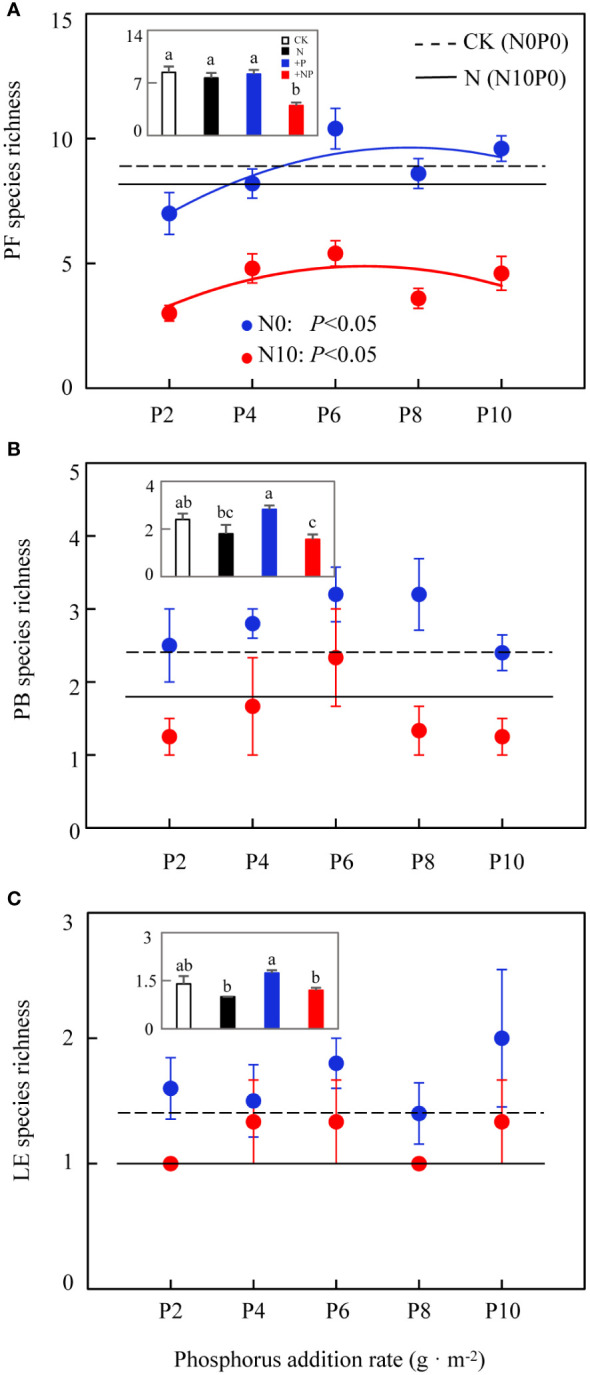
Effects of nitrogen and phosphorus addition on functional group species richness. **(A)** perennial forbs, **(B)** perennial bunch grasses, **(C)** leguminous plants. Different lowercase letters indicate significant differences at *P<* 0.05.

Two-way ANOVA revealed that nitrogen substantially affected the species richness of three functional groups (rhizomatous grasses were represented by only one species, *Leymus chinensis*, and showed no change in richness). Phosphorus only markedly influenced the species richness of forbs, with no marked effects on other functional groups (**Table 2**). The species richness of forbs was substantially affected by both nitrogen and phosphorus addition. The average species richness under CK, N, and phosphorus-only treatments was 9, 8.2, and 8.76 species, respectively; these results were substantially larger than the average of 4.28 species under combined nitrogen and phosphorus addition (**Figure 5A**, insets). In both N0 and N10, the species richness of forbs exhibited a unimodal curve with increased phosphorus addition, increasing initially and then decreasing after a threshold at 6 g P·m^-2^ (**Figure 5A**). Nitrogen addition reduced the species richness of bunch grasses (**Figures 5B**; **Table 2**), with an average species richness under CK and phosphorus-only treatments of 2.4 and 2.82 species, respectively, both of which were substantially larger than the average of 1.57 species under combined nitrogen and phosphorus addition (**Figure 5B**, insets). Nitrogen addition also reduced the species richness of leguminous plants (**Figures 5C**; **Table 2**), with the average species richness under phosphorus-only treatment (1.74 species) being substantially higher than under N (1.0 species) and combined nitrogen and phosphorus addition (1.2 species) (**Figure 5C** insets). Compared to other functional groups, the trend in species richness change for forbs was essentially consistent with the overall community species richness change, indicating that the changes in the composition of forb species were a determining factor for those in community species richness.

**Table 2:** Effects of nitrogen and phosphorus addition on functional group species richness.

Treatment	PF richness		PB richness		LE richness	
	F	*P*	F	*P*	F	*P*
N	49.170	<0.001	47.170	<0.001	29.03	<0.001
P	3.778	<0.01	1.570	0.187	0.981	0.439
N×P	1.578	0.184	1.208	0.320	0.542	0.744

(two-way ANOVA). Tabulated F and P values for comparison between groups.

### 3.4 Relationships between community species diversity and different functional group species

**Figure 6:**
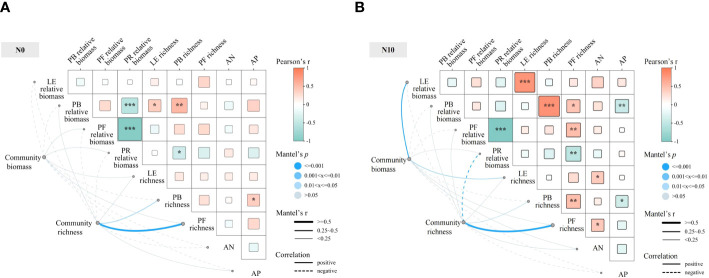
Mantel test correlation analysis of the effects of nitrogen and phosphorus on species diversity for nitrogen-limiting **(A)** and nitrogen-non-limiting **(B)** treatments. PB, perennial bunch grasses; PF, perennial forbs; PR, perennial rhizome grasses; LE, leguminous plants; AN, inorganic nitrogen; AP, available phosphorus.

Under nitrogen-non-limiting conditions, community species richness showed a highly significant, positive correlation with the richness of forbs and a strong positive correlation with the richness of bunch grasses, whereas it was strongly negatively correlated with the relative biomass of rhizomatous grasses (**Figure 6**). Community biomass was strongly positively correlated with the relative biomass and richness of leguminous plants. Among the functional groups, the species richness of forbs was strongly negatively correlated with the relative biomass of rhizomatous grasses, but positively correlated with that of other functional groups. The relative biomass of rhizomatous grasses was negatively correlated with the species richness of leguminous plants, bunch grasses, and forbs. Under nitrogen-limiting conditions, community species richness was positively correlated to a statistically significant degree with the richness of forbs and strongly positively correlated with the richness of bunch grasses. There was no statistical correlation between community biomass and the richness indices of different functional groups. Among the functional groups, the relative biomass of rhizomatous grasses was strongly negatively correlated with the relative biomass of forbs and bunch grasses.

**Figure 7:**
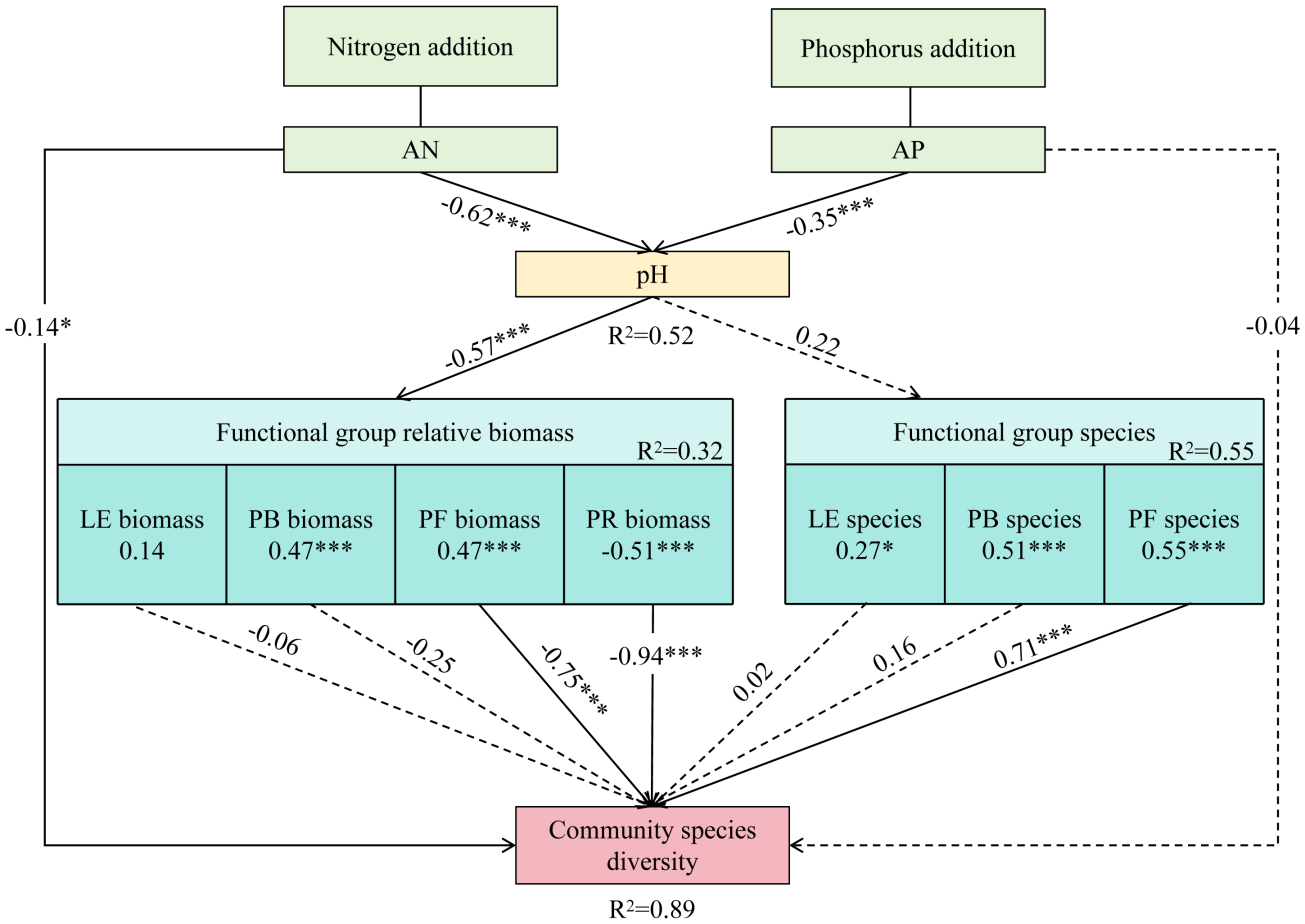
Effects of N and P Addition on grassland species diversity, as estimated by the structural equation model (SEM). Square boxes indicate variables included in the model: AN (available nitrogen); AP (available phosphorus); LE (leguminous plants); PB (perennial bunch grasses); PR (perennial rhizome grasses); PF (perennial forbs). Results of model fitting: AIC=340.260, Fisher’s C = 164.020, P=0.427. The solid line represents significant correlation. Numbers adjacent to the arrows represent standardized path coefficients.

From the structural equation model (**Figure 7**), nitrogen and phosphorus addition significantly reduced soil pH. On the one hand, soil pH was significantly positively correlated with the relative biomass of bunch grasses and forbs, and negatively correlated with that of rhizomes grasses. The relative biomass of both forbs and rhizomes grasses was negatively correlated with community species diversity. That is, the different responses of forbs and rhizomes grasses functional groups to soil pH affected community species diversity. On the other hand, pH significantly reduced community species diversity by affecting the diversity of forbs species.

## 4 Discussion

### 4.1 Impact and mechanisms of nitrogen and phosphorus addition on community species diversity

Our results show that nitrogen addition substantially reduced community species diversity, with a more pronounced reduction observed when nitrogen and phosphorus were added simultaneously. Current literature generally agrees with the conclusion that nitrogen addition reduces species diversity (Stevens et al., 2004; Midolo et al., 2019). Such as soil acidification, light competition and so on (Stevens et al., 2010). Ren et al. (2010) carried out nitrogen- and phosphorus-addition experiments in the alpine meadows of the Qinghai–Tibet Plateau and found that nitrogen addition substantially reduced community species diversity, whereas phosphorus addition had no effect. Our findings partially differ from these conclusions. In the present study, simultaneous addition of nitrogen and phosphorus substantially lowered soil pH, with even minor phosphorus addition (N10P2) causing further reduction in pH and exacerbating soil acidification, which in turn led to a decrease in species diversity (Azevedo et al., 2013). This acidification was attributed not only to increased nitrogen but also to the acidic nature of the KH_2_PO_4_ used in the experiment, as its transformation from H_2_PO_4_^-^ to HPO_4_^2-^ releases H^+^ ions. Additionally, the simultaneous addition of nitrogen and phosphorus increased the relative biomass of rhizomatous grasses, whereas it decreased that of the other three functional groups. Rhizomatous grasses gained an advantage over other plants in competing for resources, inhibiting the growth of other plants and also indirectly reducing community species diversity. Thus, under nitrogen-non-limiting conditions (N10 treatment), phosphorus addition intensified soil acidification and resource competition, leading to a greater risk of community species loss.

Under nitrogen-limiting conditions (N0 treatment), phosphorus addition had no marked effect on community species diversity compared to the control, but substantial differences were observed among different levels of phosphorus addition. Previous studies often found that phosphorus addition did not affect grassland species diversity (Ren et al., 2010), primarily because grassland plants are nitrogen-limited (Lü et al., 2016) and not sensitive to phosphorus addition (Avolio et al., 2014). However, other studies have found that the cumulative effect of phosphorus can reduce species diversity (Yang et al., 2014). An interpretation from the perspective of ecological stoichiometric homeostasis suggests that increased phosphorus causes plants to absorb more nitrogen from the soil, exacerbating nitrogen limitation and eliminating species with low nitrogen utilization efficiency. In the present study, different levels of phosphorus addition substantially influenced community species diversity. This effect might be attributed to the low available phosphorus content in the Hulunbuir Grassland (4.09 mg·kg^-1^; **Figure 1C**). At lower levels of addition (P2 and P4), competition for available phosphorus among species gradually eased (Peñuelas et al., 2012), promoting rapid growth of a larger number of forbs (**Figure 4A**). The maximum community species diversity was reached at 6 g P·m^-2^ (P6), suggesting a balanced relationship between phosphorus addition and species diversity maintenance. However, as the level of phosphorus addition increased (P8 and P10), constructive species represented by rhizomatous grasses were relieved of phosphorus limitation (**Figure 4B**), which increased their competitiveness in the community, suppressing the growth of other plants and consequently impacting community species diversity.

### 4.2 Response of species richness in different functional groups to nitrogen and phosphorus addition

Functional groups, representing collections of species with similar functional traits, exhibit consistent responses to environmental disturbances such as global change. We found that when nitrogen was not limiting, there was a notable decrease in the average species richness of each functional group. Earlier nitrogen and phosphorus addition experiments in the tallgrass prairies of the United States revealed that such additions suppressed the symbiosis between AM fungi and C_4_ grasses, consequently diminishing grass diversity and enhancing the species diversity of functional groups in forbs (Avolio et al., 2014). The present study’s findings regarding the response of grasses to nitrogen and phosphorus addition were consistent with these results. This might be attributed to the classification of grasses in the present study into rhizomatous and bunch grasses. Rhizomatous grasses consisted of only one species (*Leymus chinensis*) and were dominant, whereas the number of bunch grasses (originally three to four species) decreased due to nitrogen and phosphorus addition. This led to an overall decline in grass quantity. However, the observed response of other functional groups in the present study was generally consistent with the study of Yang et al. (2014) in the alpine meadows of the Qinghai–Tibet Plateau, who demonstrated that nitrogen and phosphorus additions increased the importance of grasses while suppressing the species diversity of forbs. This was primarily due to two reasons. First, the addition of nitrogen and phosphorus nutrients induced asymmetric competition among species (Xiao et al., 2021), favoring rhizomatous grasses in resource acquisition (e.g., nutrients and light). This was reflected in the marked increase in their relative biomass from 62.50% in the control to an average of 86.04% in various treatments, thereby inhibiting the growth of other plants. Second, nitrogen addition resulted in soil acidification, activating Al^3+^ in the soil and leading to ‘aluminum toxicity’ (Horswill et al., 2008). Grasses adapt to this type of toxicity by expanding their root sheath area to enhance tolerance, whereas forbs, lacking root sheaths, are more sensitive to Al^3+^ stress, resulting in a substantial loss of forb species (Tian et al., 2016). Additionally, the reduction in the legume functional group might be attributed to nitrogen enrichment, whereby plants can directly utilize exogenous available nitrogen and thus reduce the reliance on their own nitrogen fixation processes. This disrupts the symbiotic relationship with mycorrhizal fungi, subsequently impacting the absorption of other nutrients and leading to nutrient limitations, and further restrictions on the growth of leguminous plants (Lan and Bai, 2012).

In contrast to nitrogen addition, which reduced species richness across various functional groups, phosphorus addition under nitrogen limitation substantially affected only the forb functional group. This process was characterized by an increase in species richness at lower levels of phosphorus addition and a decrease at higher levels. The substantial impact of phosphorus addition on the species richness of forbs is attributable to two facts: (1) the number of forb species in the study region was high, leading to greater impact of phosphorus addition; (2) the available phosphorus content in the Hulunbuir Grassland was low (see Section 3.1). In contrast, the impact of phosphorus on bunch grasses and leguminous plants was not substantial, likely because their species abundance was low, or because the study duration was insufficient to fully manifest ecological effects.

### 4.3 Correlation analysis of components causing changes in community composition

We observed an inverse trend between the relative biomasses of forbs and rhizomatous grasses. An increase in the relative biomass of rhizomatous grasses, limiting the growth of forbs, was a key factor in the reduction of community species diversity. Under nitrogen-non-limiting conditions, community species richness exhibited a strong positive correlation with the species richness of bunch grasses and forbs, but a strong negative correlation with the relative biomass of rhizomatous grasses. When combined with the observed decrease in community species richness, these findings indicate that the importance of rhizomatous grasses in regulating changes in community composition. As noted above, the increase in the relative abundance of dominant species due to nitrogen deposition is a primary driver for the loss of species diversity in grassland communities under nitrogen deposition, with forbs showing no strong competitive interactions with other species. The species richness of forbs, leguminous plants, and bunch grasses, as three functional groups, was positively correlated with community species richness, further confirming that the strengthening of the dominance of already-dominant species is the main mechanism leading to the loss of species diversity in grassland communities under nitrogen deposition.

## 5 Conclusion

The addition of nitrogen and phosphorus nutrients is known to impact grassland community species diversity and alter the species composition of different functional groups. Our study found that under nitrogen-non-limiting conditions in the Hulunbuir meadow steppe, local species diversity decreased and the addition of phosphorus further increased the risk of species loss. Under nitrogen-limited conditions, species diversity was not substantially affected by phosphorus addition compared to the control but showed substantial differences at various levels of phosphorus addition, forming a unimodal curve. Under both nitrogen-limiting and non-limiting conditions, species diversity reached a turning point at a threshold of 6 g·m^-2^ of phosphorus addition. Leguminous plants, bunch grasses, and forbs showed a consistent response to nitrogen and phosphorus addition, in contrast to rhizomatous grasses, which were the main species driving community composition changes. Phosphorus addition intensified the risk of nitrogen deposition-induced species loss. Soil acidification and the increased dominance of already-dominant species lead to species loss in the forb functional group, which is the primary mechanisms for the decline in community species diversity. However, the nutrient addition effects were considerably influenced by environmental factors such as precipitation, indicating that some conclusions may require further validation using long-term data.

## Data availability statement

The original contributions presented in the study are included in the article/supplementary material. Further inquiries can be directed to the corresponding author.

## Author contributions

ZW: Visualization, Writing – original draft, Data curation, Investigation. LC: Investigation, Writing – review & editing. YP: Investigation, Writing – review & editing. DZ: Data curation, Writing – review & editing. YY: Formal Analysis, Writing – review & editing. XL: Data curation, Writing – review & editing. HW: Writing – review & editing, Funding acquisition.
